# Rethinking Parent-Mediated Intervention in Early Autism: A Model for Developmental, Relational, and Scalable Care

**DOI:** 10.1192/bjo.2026.11074

**Published:** 2026-06-30

**Authors:** Leanne Kade, Becky MacRitchie, Henry Needham

**Affiliations:** 1 Milton Keynes University Hospital NHS Foundation Trust, Milton Keynes, United Kingdom; 2 Norwich Medical School, University of East Anglia, Norwich, United Kingdom

## Abstract

**Aims:**

This integrative thematic synthesis aims to synthesise evidence on (1) neurodevelopmental and relational mechanisms hypothesised to drive Parent-Mediated Intervention effects; (2) lived experience and acceptability of parents, and (3) feasibility of widespread implementation into early-years services. Results were shaped into a translational model to inform sustainable, scalable, and ethical early-intervention delivery.

**Methods:**

A scoping review was conducted in-line with PRISMA-ScR guidance. The databases Medline, PubMed, Scopus, and PsycINFO were searched for studies published between 2010 and 2025. Eligible papers included infants up to 24 months old at increased risk of autism and reported on at least one of the following areas: proposed neurodevelopmental mechanisms, parental experience, or implementation and scalability. Two reviewers independently screened and synthesised data from qualitative studies, clinical trials, and service evaluations using thematic analysis and cross-domain integration.

Studies were excluded if they focused solely on pharmacological interventions or diagnostic procedures, as well as those only including children older than 24 months.

Studies that were not addressing the practical delivery or feasibility of the intervention within real-world service settings were also excluded. This approach enabled inclusion of both trials examining efficacy and underlying mechanisms, alongside research exploring lived experience and system-level implementation.

**Results:**

The thematic analysis identified three interconnected domains: (1) Parent-Mediated Intervention shapes early social attention and arousal regulation by increasing the predictability of caregiver social signals without altering core neuro development trajectories; (2) parental empowerment and stress regulation act as key mediators of sustained engagement and parent–infant synchrony; and (3) feasibility and equity are determined by delivery model, workforce capacity, accessibility and adaptability (for example, hybrid or telehealth models).

Heterogeneity in terminology, study design, intervention duration, and outcome measures (including criteria used to define autism likelihood) was acknowledged as a limitation constraining synthesis across the studies in this review.

Despite these limitations, findings were integrated into a Developmental-Relational-Implementation model to support clinicians and commissioners in selecting, timing, and delivering Parental-Mediated Interventions that are feasible and adaptable within real-world health systems.

**Conclusion:**

The Developmental-Relational-Implementation model offers a pragmatic framework for clinicians, commissioners, and policymakers to guide the delivery of Parental-Mediated Intervention in early autism care. This review positions Developmental-Relational-Implementation-informed approaches as family-centred, equitable, and scalable by capitalising on relational engagement, developmental neuroplasticity, and the practical realities of healthcare delivery. To support scalable and sustainable expansion, furtherresearch should incorporate mechanistic markers, qualitative assessments of parental outcomes, and health economic evaluation.

